# Multilocus sequence analyses reveal extensive diversity and multiple origins of fluconazole resistance in *Candida tropicalis* from tropical China

**DOI:** 10.1038/srep42537

**Published:** 2017-02-10

**Authors:** Jin-Yan Wu, Hong Guo, Hua-Min Wang, Guo-Hui Yi, Li-Min Zhou, Xiao-Wen He, Ying Zhang, Jianping Xu

**Affiliations:** 1 Laboratory for Conservation and Utilization of Bio-Resources, and Key Laboratory for Microbial Resources of the Ministry of Education, Yunnan University, Kunming, Yunnan, China; 2 Public Research Laboratory, Hainan Medical University, Haikou, Hainan, China; 3 Department of Biology, McMaster University, Hamilton, Ontario, L8S 4K1, Canada

## Abstract

*Candida tropicalis* is among the most prevalent human pathogenic yeast species, second only to *C. albicans* in certain geographic regions such as East Asia and Brazil. However, compared to *C. albicans*, relatively little is known about the patterns of genetic variation in *C. tropicalis*. This study analyzed the genetic diversity and relationships among isolates of *C. tropicalis* from the southern Chinese island of Hainan. A total of 116 isolates were obtained from seven geographic regions located across the Island. For each isolate, a total of 2677 bp from six gene loci were sequenced and 79 (2.96%) polymorphic nucleotide sites were found in our sample. Comparisons with strains reported from other parts of the world identified significant novel diversities in Hainan, including an average of six novel sequences (with a range 1 to 14) per locus and 80 novel diploid sequence types. Most of the genetic variation was found within individual strains and there was abundant evidence for gene flow among the seven geographic locations within Hainan. Interestingly, our analyses identified no significant correlation between the diploid sequence types at the six loci and fluconazole susceptibility, consistent with multiple origins of fluconazole resistance in the Hainan population of *C. tropicalis*.

The yeast genus *Candida* is broadly distributed in a diversity of ecological niches, including soil, plant materials, animals, and the human oral mucosa and other body surfaces. With the increasing number of immunocompromised patients including cancer and organ transplant patients as well as the widespread use of broad-spectrum antibiotics, *Candida* has emerged as a major group of opportunistic pathogens that can cause serious invasive infections^1^,^2^,^3^. Invasive infections caused by *Candida* yeasts have been associated with significant morbidity and mortality^4^,^5^,^6^,^7^. Although *Candida albicans* is the most prevalent opportunistic yeast pathogen, other non-*albicans Candida* species such as *C. tropicalis* are also commonly found and their frequencies have increased steadily in recent years. In certain geographic regions such as East Asia and Brazil, *C. tropicalis* is the first or second most prevalent pathogenic yeast species^8^,^9^,^10^,^11^,^12^,^13^,^14^. However, compared to *C. albicans*, relatively little is known about the molecular epidemiology of *C. tropicalis* in many regions around the world, including tropical Asia.

Over the past two decades, many molecular typing methods have been used to identify genotypes and examine the relationships among strains of pathogenic yeasts. Similar to *C. albicans, C. tropicalis* is a diploid yeast and is evolutionary closely related to *C. albicans*^15^. The diploid nature can make its genotyping difficult to score as dominant markers such as PCR fingerprinting profiles and amplified fragment length polymorphisms often can’t distinguish homozygotes from heterozygotes^16^,^17^. For *C. tropicalis,* the emerging consensus since 2005 for strain typing is multilocus sequence typing (MLST), which is based on the analysis of single nucleotide polymorphisms (SNPs) at six gene fragments^18^. These co-dominant markers have been found to be highly polymorphic and discriminatory and they have been used to monitor strain maintenance, replacement, and microevolution within human hosts^19^,^20^,^21^,^22^,^23^. The establishment of a MLST database for *C. tropicalis* (as well as databases for other common pathogenic microbes at pubmlst.org) has facilitated the comparisons of strains and populations from different laboratories and different geographic regions in the world^24^. The current MLST database for *C. tropicalis* includes DNA sequence information at the following six loci (*ICL1, MDR1, SAPT2, SAPT4, XYR1* and *ZWF1a*) for over 600 isolates from Europe, Asia, and the Americas^18^,^19^,^20^,^21^,^22^,^23^.

Given the high prevalence of *C. tropicalis* in tropical regions and its increasing medical significance, it’s important to understand the patterns of genetic variation of this yeast species in the tropics. Here in this study, we analyzed strains of *C. tropicalis* from the tropical island of Hainan in southern China. Hainan Island is located in China’s southernmost province, Hainan Province (latitude 3°30′–20°10′N; longitude 108°15′–120°15′ E). The island is separated from Mainland China by Qiongzhou Strait, ~40 km in width. Similar to those found in several other tropical regions, recent epidemiological analyses of yeasts in Hainan identified that *C. tropicalis* is among the most common yeast species from the oral cavities of asymptomatic hosts, second only to *C. albicans*^14^,^25^. Interestingly, a number of *C. tropicalis* strains from hosts in Hainan not exposed to fluconazole showed resistance and tolerance to the most common antifungal drug fluconazole^25^. At present, the patterns of genetic variation among strains and geographic populations and the genetic relationships between fluconazole-resistant and fluconazole-susceptible isolates from Hainan are unknown.

The objective of this study is to analyze the patterns of genetic variation of *C. tropicalis* from Hainan Island. Because Hainan is a tropical island with abundant organic matter conducive for the growth of *C. tropicalis*, we hypothesize that the favorable environmental conditions for *C. tropicalis* in Hainan may allow the generation and maintenance of abundant genetic variation of this species within Hainan. Furthermore, since human colonization of the Island is relatively recent in evolutionary terms and that there is frequent migration of people among different regions within the island, especially since the 1980s, we further hypothesize that populations of *C. tropicalis* from different geographic regions of the Island should be highly similar to each other. Finally, our recent study identified unexpected resistance and tolerance to fluconazole among isolates of *C. tropicalis* from this island, we were interested in whether there is any association between strain MLST genotype and drug resistance pattern.

## Results

We successfully obtained DNA sequences from all six loci (*ICL1, MDR1, SAPT2, SAPT4, XYR1* and *ZWFa1*) for all 116 isolates from 7 regions in Hainan (Tables 1 and 2). The genomic locations of these six loci are presented in Supplementary Table 1. As shown in this table, these six loci are situated on six different supercontigs corresponding to six chromosomal scaffolds. A total of 2677 bp from the six gene loci were sequenced for each of the isolates and 79 (2.96%) polymorphic nucleotide sites were found among our strains. All six gene fragments were found to be polymorphic among isolates within the Hainan population of *C. tropicalis*. The number of sequence types at each gene fragment ranged from 11 to 37, with a mean of 26.6 sequence types per gene fragment among the 116 isolates. Among the combined total of 124 sequence types at the six gene fragments, 36 were found to be new and had never been reported from other geographic regions. The combined analyses of sequence information from the six gene fragments identified a total of 94 diploid sequence types (DSTs) (Table 2). Among these 94 DSTs, only 14 have been reported previously and the remaining 80 DSTs were completely new to the database. The details about the genetic variation at each of the six gene fragments are briefly described below.

ICL1. 

Of the 447 aligned nucleotides of the *ICL1* locus, 6 were found to be variable in the Hainan population of *C. tropicalis*. The 6 SNP sites generated a total of 8 genotypes (Table 3) among the 116 isolates from Hainan. Among these 8 genotypes, 7 (representing 114 isolates) have been reported from outside of Hainan while the remaining one (representing 2 isolates Ct_C14 and Ct_C20) is new, so far found only in Hainan. However, this novel genotype was clustered with known genotypes in the database from other geographic locations. The relationships among our genotypes and representatives of the unique genotypes at the *ICL1* locus in the MLST database are shown in Supplementary Figure 1. The most frequent genotype at this locus, genotype 1, was found in 86 of the 116 isolates (74.1%).

### MDR1

Of the 425 aligned nucleotides of the *MDR1* locus, 21 were found to be variable in the Hainan population of *C. tropicalis*. These 21 SNP sites generated a total of 37 genotypes at this locus (Table 3) among the 116 isolates from Hainan. Among these 37 types, 23 (representing 97 isolates) have been previously reported from outside of Hainan while the remaining 14 (representing 19 isolates) are so far found only in Hainan. The 14 novel genotypes contained both closely related (e.g. strain Ct_BT107) and moderately related (e.g. Ct_SY15) ones to those present in the existing MLST database. The relationships among our genotypes and representatives of the unique genotypes at the *MDR1* locus in the MLST database are shown in Supplementary Figure 2. The most frequent genotype 9 was found in 23 of the 116 isolates (19.8%) and the second most frequent was genotype 22, found in 18 isolates (15.5%).

### SAPT2

Of the 525 aligned nucleotides of the *SAPT2* locus, 7 were found to be variable in the Hainan population of *C. tropicalis.* These 7 SNP sites generated a total of 11 genotypes at this locus (Table 3) among the 116 isolates from Hainan. Among these 11 types, 7 (representing 109 isolates) have been previously reported from outside of Hainan while the remaining 4 (representing 7 isolates) are so far found only in Hainan. The 4 novel genotypes were all closely related to those in the existing MLST database. The relationships among our genotypes and representatives of the unique genotypes at the *SAPT2* locus in the MLST database are shown in Supplementary Figure 3. The most frequent three genotypes are genotype 1 (found in 31 isolates, 26.7%), genotype 3 (found in 34 isolates, 29.3%), and genotype 12 (found in 35 isolates, 30.1%).

### SAPT4

Of the 390 aligned nucleotides of the *SAPT4* locus, 18 were found to be variable in the Hainan population of *C. tropicalis*. These 18 SNP sites generated a total of 21 genotypes at this locus (Table 3) among the 116 isolates from Hainan. Among these 21 types, 16 (representing 107 isolates) have been previously reported from outside of Hainan while the remaining 5 (representing 9 isolates) are so far found only in Hainan. The 5 novel genotypes at this locus were all closely related to those present in the existing MLST database. The relationships among our genotypes and representatives of the unique genotypes at the *SAPT4* locus in the MLST database are shown in Supplementary Figure 4. The most frequent genotype, genotype 7, was found in 28 isolates (24.1%) and the second most frequent genotype 17 was found in 26 isolates (22.4%).

### XYR1

Of the 370 aligned nucleotides of the *XYR1* locus, 16 were found to be variable in the Hainan population of *C. tropicalis*. These 16 SNP sites generated a total of 33 genotypes at the locus (Table 3) among the 116 isolates from Hainan. Among these 33 types, 25 (representing 106 isolates) have been previously reported from outside of Hainan while the remaining 8 (representing 10 isolates) are so far found only in Hainan. The 8 novel genotypes contained both closely related (e.g. strain Ct_BT107) and moderately related (e.g. Ct_NK211 and Ct_1_HNHK) ones to those present in the existing MLST database. The relationships among our genotypes and representatives of the unique genotypes at the *XYR1* locus in the MLST database are shown in Supplementary Figure 5. The most frequent genotype, genotype 60, was found in 26 isolates (22.4%) and the second most frequent genotype 9 was found in 15 isolates (12.9%).

### ZWFa1

Of the 520 aligned nucleotides of the *ZWFa1* locus, 11 were found to be variable in the Hainan population of *C. tropicalis*. These 11 SNP sites generated a total of 14 genotypes at this locus (Table 3) among the 116 isolates from Hainan. Among these 14 types, 10 (representing 112 isolates) have been previously reported from outside of Hainan while the remaining 4 (representing 4 isolates) are so far found only in Hainan. The four novel genotypes at this locus were all closely related to those present in the existing MLST database. The relationships among our genotypes and representatives of the unique genotypes at the *ZWFa1* locus in the MLST database are shown in Supplementary Figure 6. The most frequent genotype, genotype 22, was found in 35 isolates (30.1%) and the second most frequent genotype 3 was found in 28 isolates (24.1%).

### The combined diploid sequence types based on all six gene fragments

The combined analyses of all six gene fragments identified that the 116 isolates from Hainan contained 94 diploid sequence types (DSTs). Even though a significant proportion of the alleles at each locus were shared between Hainan and outside of Hainan, relatively few combined DSTs at the six loci (14/94) were shared between the Hainan population and those from other parts of the world. The shared DSTs and the specific numbers of strains from within Hainan for each of these DSTs are DST149 (5 strains), DST331 (4 strains), DST346 (3 strains), DST394 (7 strains), DST427 (2 strains), DST430 (4 strains), DST432 (2 strains), DST 465 (2 strains), and DST490 (2 strains). The remaining 85 known DSTs have one strain each in our analyzed Hainan sample.

**Figure 1:**
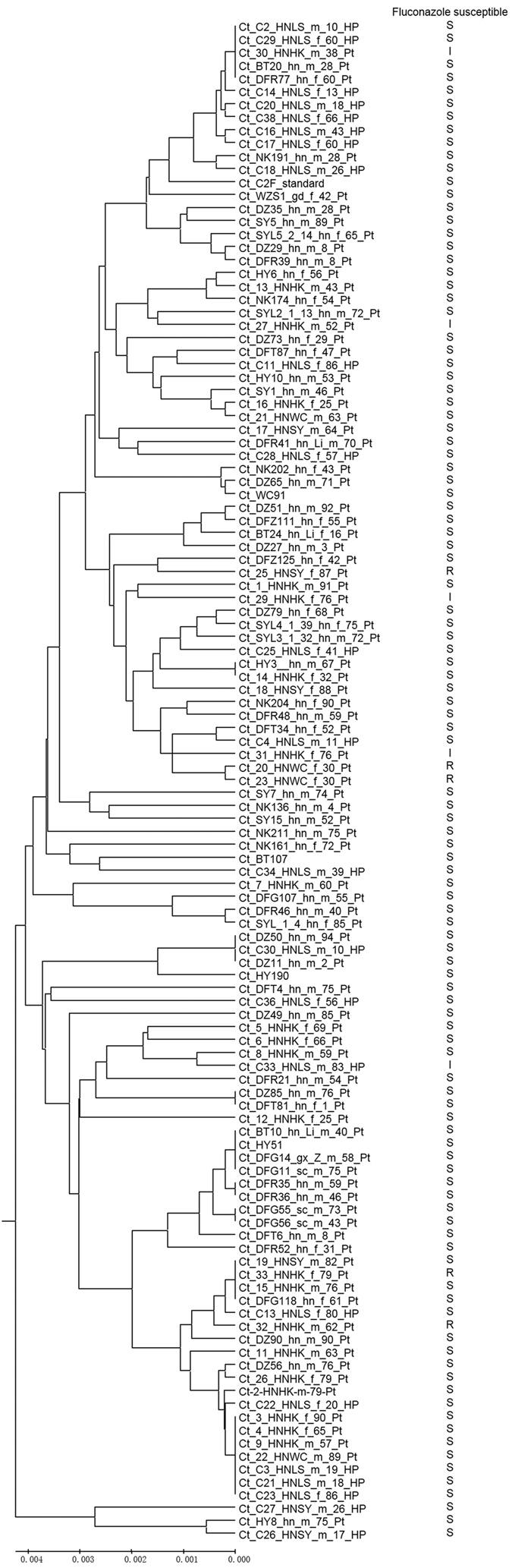
UPGMA dendrogram showing genetic similarities among 116 *C. tropicalis* isolates from Hainan as determined by MLST of six gene loci.

Among the 14 shared DSTs between Hainan and outside of Hainan, nine were shared with those from Mainland China, two were shared with those from Taiwan, one each from Korea and the Netherlands. The remaining one shared DST was found in multiple countries/regions. The genetic relationships among the 116 isolates based on sequences at all six loci are shown in Fig. 1.

### Evidence for extensive gene flow among geographic populations

The population genetic analyses of our samples based on nucleotide information at the 79 polymorphic nucleotide sites from the six gene fragments revealed that the majority (75%) of the genetic variation was found within individual strains (Table 4). The second most important contributor was the differences among individuals within individual geographic populations that contributed 23% of the total genetic variation. In contrast, the geographic separations among local and regional populations contributed relatively little to the overall patterns of genetic variation (Table 4). In addition, multiple DSTs were shared among regions within Hainan (Table 2). Together, the presence of shared DSTs and the lack of genetic differentiation among geographic populations of *C. tropicalis* within Hainan are consistent with frequent gene flow among these regions in Hainan.

**Figure 2:**
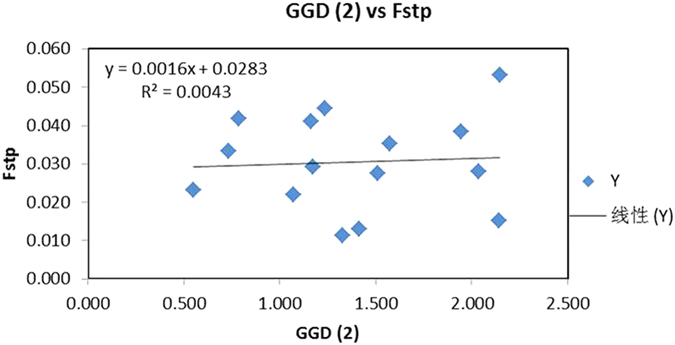
A Mantel test between Nei’s genetic distance and the two-dimensional geographical distances (based on longitudinal and latitudinal coordinates) among populations. No significant correlation was found between the two variables (p = 0.390).

Aside from conducting the overall AMOVA, we also obtained the F_ST_ values between pairs of geographical populations. However, to ensure the robustness of the results, the populations of less than five samples were removed in the pairwise comparisons. This analysis identified no statistically significant differentiation between any pairs of geographic populations. The lowest F_ST_ value (0.011) was found between Haikou and Lingshui while the highest (0.053) was between Wenchang and Sanya (Table 5). The result from the Mantel test is shown in Fig. 2. The test showed that there was little correlation between genetic distance and geographical distance among the analyzed populations (P = 0.390), consistent with extensive gene flow among the geographic populations.

### eBURST analysis

We used the eBURST program to identify genotype clusters^26^. In this analysis, we applied the default setting of identical alleles at five of the six loci for genotype cluster identification and 1000 re-samplings for confidence estimates through bootstrapping. Among the 502 DSTs from the C. *tropicalis* MLST database, the eBURST analysis found 55 clusters and 214 solitary DSTs, known as singletons. Of the 94 DSTs representing the 116 isolates in our sample from Hainan, 50 DSTs were grouped into 20 clusters (clusters 1, 2, 4, 5, 7, 10, 11, 14, 18, 23, 32, 34, 44, 46, 47, 48, 49, 50, 51 and 53), and 44 DSTs were classified as singletons (i.e. not belonging to any obvious clusters) (Table 2). Specifically, cluster 1 had a total of 31 DSTs, including 2 from this study. Cluster 2 had a total of 21 DSTs, with 1 from this study. Cluster 3 had a total of 14 DSTs, including one from this study. Cluster 4 had a total of 17 DSTs, including 14 from this study. The 14 Hainan DSTs in cluster 4 contained a total of 28 isolates distributed in all seven geographic regions. Among these 14 DSTs, DST 394 was represented by 7 isolates from three regions in Hainan (Haikou, Lingshui, and Wenchang) and occupied the central position of this cluster. DSTs 331 and 430 had four strains each with each DST distributed in three regions respectively (Table 2).

Another cluster with multiple DSTs and multiple strains from Hainan was cluster 7. This cluster had a total of 11 DSTs, including 8 from this study. Among these eight DSTs, one (DST 149) was at the center of this cluster and it contained five isolates from four regions in Hainan (Baoting, Dongfang, Haikou, and Lingshui). Two isolates in this cluster have intermediate resistance to fluconazole (see also below). Cluster 10 had a total of 8 DSTs, including 3 from this study with isolate C30 representing DST 333 at the center of this cluster. Cluster 11 had a total of 7 DSTs, including one from this study. Cluster 14 had a total of 5 DSTs, including one from this study. Clusters 23 and 34 each had 3 DSTs while Clusters 44, 46 and 53 each had 2 DSTs. Each of these five clusters (i.e. clusters 23, 34, 44, 46, and 53) contained only one strain each from Hainan. Clusters 18, 47, 48, 49, 50 and 51 are new clusters added from this study and they comprised DSTs identified only in the present work. Among the singletons, isolates Ct_HY8, Ct_C26P, and Ct_C27 showed the largest genetic distances from the other DSTs. Overall, results from the eBURST analyses were consistent with the UPGMA tree generated from multilocus sequence data and demonstrated that the genotypes from Hainan were dispersed in most of the known clusters that included strains from other geographic areas (Fig. 1).

### Fluconazole susceptibility analysis

Among these 116 isolates, four were resistant to fluconazole, five had dose-dependent intermediate resistance to fluconazole, and the remaining 107 were susceptible to fluconazole. The nine fluconazole dose-dependent/resistant isolates belonged to nine different DSTs (DST 149, 331, 416, 436, 441, 474, 477, 478, and 500; Table 2, Fig. 1). Four (DST 149, 331, 441, and 500) of the 9 DSTs belong to 3 genotype clusters while the remaining five were singletons (Table 2). Among these nine DSTs, DST 331 and DST 149 also contained fluconazole susceptible strains. Specifically, fluconazole-susceptible isolates Ct_BT20, Ct_C2, Ct_C29, and Ct_DFR77 shared the same DST 149 with fluconazole dose-dependent isolate Ct_30_HNHK (Fig. 1). Similarly, fluconazole-susceptible isolate Ct_15_HNHK, Ct_19_HNSY and Ct_DFG118 shared the same DST331 with the fluconazole-resistant isolate Ct_33_HNHK (Fig. 1). These dose-dependent/resistant strains were distributed broadly among the clusters and across the UPGMA tree. Taken together, our results suggest that fluconazole resistance among Hainan *C. tropicalis* isolates most likely originated multiple times through independent mutations.

**Figure 3:**
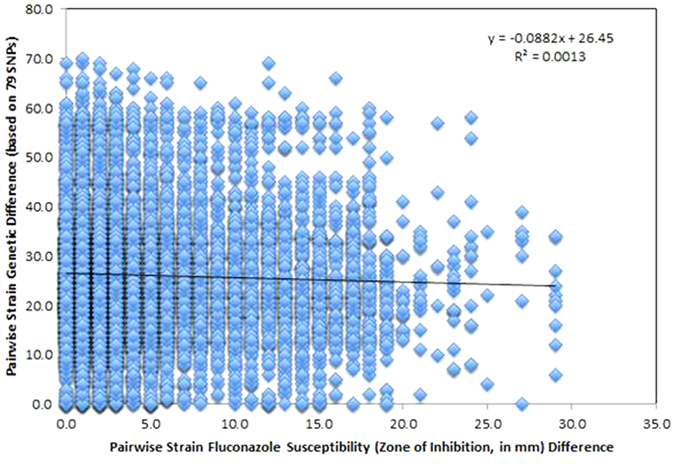
A Mantel test between genetic difference (based on 79 SNPs) and fluconazole susceptibility (zone of inhibition, in mm) among strains. No significant correlation was found between the two variables (P = 0.238).

The independent origin hypothesis for fluconazole resistance is also supported by the Mantel test results (Fig. 3). Specifically, our analyses showed that pairwise strain genetic distance and fluconazole susceptibility differences were not correlated with each other (p = 0.238). This result is consistent with not only the independent origins of fluconazole resistance among the strains analyzed here but also the hypothesis that many, if not all, genotypes or genotype clusters are capable of developing fluconazole resistance.

## Discussion

This study analyzed the patterns of DNA sequence variation at six nuclear gene loci and compared the patterns of variation with those in the MLST database representing strains from other geographic regions. Our analyses revealed extensive novel sequence polymorphisms not only at the individual locus level but also more noticeably at the combined genotype level. Interestingly, most genetic variations were found within individual strains and among strains within the same geographic populations. Despite the extensive genetic variations within individual populations, we found no evidence of genetic differentiations among the analyzed geographic populations within Hainan, consistent with frequent gene flow among these geographic regions. In addition, even though evidence for clonal dispersal and expansion were found in our samples for fluconazole-susceptible genotypes, there was no evidence of clonal dispersal for fluconazole-resistant isolates. Each of the nine fluconazole-resistant or dose-dependent isolates belonged to a different multilocus DST. Among these nine DSTs, three also had representatives of fluconazole-susceptible strains, consistent with the independent mutations causing fluconazole resistance among our nine strains. However, the lack of evidence for clonal expansion of fluconazole-resistant genotypes in Hainan doesn’t mean that such clonal expansions do not exist at all in Hainan. Indeed, the inclusion of more strains from Hainan, especially those that are resistant to fluconazole, might reveal clonal expansion of fluconazole-resistant *C. tropicalis* in Hainan. Below we discuss the implications of our results.

Our study is the first genetic analysis of *C. tropicalis* from tropical Asia. The genetic variations observed here expand our understanding of this organism in nature. However, even though we identified abundant genetic variations, we believe that there are likely additional genetic variations in Hainan and in other parts of tropical Asia. For example, there might be bias in the efficiency of PCR amplification between the two alleles at each heterozygous locus (e.g. due to mutations in primer sequences between the two alleles) that could have resulted in underestimates of heterozygosity within individual strains. Furthermore, our samples were all from one ecological niche, the oral cavities of humans. *C. tropicalis* has been found in a diversity of other environments, including organically enriched soil and aquatic environments^17^,^27^, and animals such as wild birds^28^, horses^29^, rheas^30^ as well as in tortoises and sea turtles^31^. Hainan Island and the tropics in general are rich in organic compounds and wild animals. Thus, it’s possible that these ecological niches contain additional genetic diversity of *C. tropicalis* and one or several of these niches may represent the natural reservoirs of *C. tropicalis* for humans. A population genetic comparison of *C. tropicalis* from these environments with our data here could reveal the relationships between these populations and help identify the environmental reservoir(s) of *C. tropicalis* in Hainan (as well as elsewhere).

The potential existence of an environmental reservoir of *C. tropicalis* for humans is also supported by our data. Specifically, none of the hosts had taken any fluconazole or other triazole drugs. However, nine *C. tropicalis* isolates from nine different hosts showed intermediate susceptibility or were resistant to fluconazole. We believe the likely source(s) for the observed fluconazole resistance in *C. tropicalis* is natural or human-made environments in Hainan. As shown recently in another opportunistic human fungal pathogen *Aspergillus fumigatus*, the application of agricultural fungicides was most likely responsible for the emergence of drug-resistant strains in human populations for that filamentous fungus^32^,^33^,^34^. A similar process could have happened here whereby drug-resistant strains selected in agricultural fields with heavy applications of triazole fungicide were passed onto human hosts^35^. Alternatively, other types of settings, e.g. human-made products such as paint or human-associated environments such as house stuff, where antifungal agents are applied could also select for drug-resistant fungal strains. Targeted samplings of agriculture fields or other environments where triazole fungicides are commonly used could help reveal the potential sources of fluconazole-resistance in *C. tropicalis* in Hainan.

Regardless of the potential sources, such environmentally induced drug-resistance isolates pose a significant threat to human and animal health. This can be especially troublesome for tropical regions where *C. tropicalis* candidaemia are of particular concern^3^. Patients infected with triazole-resistant C. *tropicalis* are often associated with high mortality^3^,^4^,^9^,^17^,^29^. In this study, all the C. *tropicalis* strains were isolated from oral cavities of local healthy people or in-patients in hospitals. The oral cavity is a significant niche of the human microbiome and a gateway for the microbiota in many other human body sites. A drug-resistant strain from the oral cavity could be passed on to other body sites, potentially causing untreatable invasive infections.

The six gene fragments analyzed here showed abundant genetic variation within and among strains from Hainan (Table 3). The number of polymorphic sites (79 of 116 isolates) in the present study is slightly higher than that (28 of 58 isolates) from Beijing, China, but lower than those in other places. For example, the number of polymorphic sites from Brazil was 154 among 61 isolates (Table 3). In contrast, except for the *XYR1* locus, the ratios for the number of genotypes per polymorphic nucleotide site in our samples are higher than previously reported for geographic populations including the US and European countries and Brazil (Table 3). These ratios were slightly different for the Beijing sample where extremely large ratios were found, e.g. one polymorphic nucleotide site allowed the identification of 15 genotypes at the *XYR1* locus^22^. Specifically, if this polymorphic site is biallelic (i.e. containing two alternative bases), a maximum of 3 genotypes should be found (two homozygotes and one heterozygote). With three alternative bases, a maximum of 9 genotypes would be expected in a diploid organism at this site. Only with all four alternative bases at this site in the Beijing sample would we expect a maximum of 16 genotypes based on one polymorphic nucleotide site and assume all possible associations among the four bases at this site in this diploid organism.

Interesting, the most frequent genotypes in our sample at *ICL1* (genotype 1), *SAPT2* (genotype 3) and *SAPT4* (genotype 7) were also the most frequent in the global population analyzed so far. In contrast, the most frequent genotype at the other three loci *MDR1* (genotype 9), *XYR1* (genotype 60), and *ZWFa1* (genotype 22) in Hainan were not the most frequent in the global population analyzed so far. Furthermore, the most frequent genotype at five of the six loci (except *ICL1*) in our samples also differed from that reported from Beijing. China. Specifically, the most frequent genotypes at *MDR1, SAPT2, SAPT4, XYR1,* and *ZWFa1* from the Beijing sample were genotype 7, 4, 17, 2 and 7 respectively^22^. Together, these data suggested that the Hainan *C. tropicalis* population contained abundant and novel genetic variation at the assayed loci.

The observed novel genetic variation was found not only at the individual locus level. At the combined DST level from all six sequenced gene fragments, only 14 of the 94 DSTs from the 116 strains were shared with those from other geographic areas while the remaining 80 were novel to the C. *tropicalis* MLST community database. Among the 14 shared DSTs between Hainan and those from outside of Hainan, 13 were shared with strains from within east Asia, including nine DSTs (i.e. DSTs 330, 331, 333, 336, 343, 346, 348, 351 and 374) from Mainland China^20^,^22^,^23^, three from Taiwan (DSTs 149, 197, and 203)^19^, and one (DST394) from Korea. Only one DST (DST 23) was shared only with a strain from outside of Asia (the Netherlands)^18^. DST 23 is a singleton genotype in the MLST database and the carrier of this strain in Hainan was an 11 year-old schoolboy in Lingshui along the east coast of Hainan. He had no travel history to the Netherlands. Interestingly, DST203 found on both Hainan Island and Taiwan Island has also been found in Brazil^21^. Together, these results suggest the potential of long distance dispersal for *C. tropicalis* among geographic regions, likely through humans or human activities, including importing and exporting of foods colonized by *C. tropicalis*.

Among these shared DSTs, one (DST149) is worthy of special mention. DST 149 was represented by fluconazole resistant strains in both Taiwan Island and Hainan Island^36^,^37^. Furthermore, DST 149 was the main fluconazole-resistant DST in Taiwan from 1999–2006^36^. Thus, instead of independent origins, it’s possible that the Hainan fluconazole-resistant strain of *C. tropicalis* could have originated in Taiwan and dispersed to Hainan (or vice versa). However, more strains need to be investigated from Southeast Asia, including from southern China and the Philippines, before the conclusion about a Taiwan-Hainan transfer of this specific fluconazole-resistant genotype could be made.

While our results showed no genetic clustering of fluconazole resistant isolates, possibly due to the high-level genotype diversity and the relatively limited sampling in our study, several studies of similar or smaller sample sizes found evidence of genetic clustering of azole-resistant isolates. For example, both Chou *et al*.^19^ and Li *et al*.^35^ found clonal cluster 2 (containing DST140 and DST98) was enriched with isolates with resistance or trailing growth in the presence of fluconazole. A recent report by Wang *et al*.^38^ showed that 23 of the 30 azole-resistant isolates of *C. tropicalis* from Shanghai belonged to four DSTs (DST 376, 505, 506, and 507) of the same genetic cluster. However, though four DSTs (DST 149, 331, 346, and 394) were shared between our sample and those in the recent Wang *et al*. study, none of these four DSTs had fluconazole-resistant isolates in both the Shanghai and Hainan samples. Similarly, Chen *et al*.^20^ found that DST 164 was associated with a high MIC to flucytosine. Clonal expansion of flucytosine resistance in *C. tropicalis* has also been reported from Paris, France^39^. Furthermore, Li *et al*.^36^ described several single locus genotypes (genotype #3 of *ICL1*, #9 of *MDR1*, #1 of *SAPT2*, #3,6 and 10 of *SAPT4*, #48 of *XYR1* and #7 of *ZWF1a*) associated with low MICs to fluconazole. Different from these studies, we found no isolate with these seven DSTs (DST 98,140, 164, 376, 505, 506, and 507) in our sample. In addition, genotype #3 of *ICL1*, #1 of *SAPT2,* and #7 of ZWF1a were found associated with both fluconazole-susceptible and fluconazole-resistant isolates in Hainan (Table 2 and Fig. 2). Together, these results suggest both shared and unique features among geographic populations of *C. tropicalis*.

At present, the molecular mechanisms of resistance among our strains are not known. Though we found evidence for multiple independent origins of the fluconazole-resistant strains in our samples, their mechanisms of resistance could be very similar or even identical. Molecular studies of triazole-resistant strains of *Candida* have shown three common types of mechanisms: (i) mutation of the target gene *ERG11* (or *CYP51*) leading to reduced affinity of the drugs to the target enzyme; (ii) over-expression of *ERG11*; and (iii) over-expression of efflux pumps^40^. For example, Barchiesi *et al*.^41^ found that over expressions of the major facilitator gene *MDR1* and the ATP-binding cassette transporter *CDR1* were responsible for fluconazole resistance among independently selected fluconazole-resistant mutants of *C. tropicalis* strain ATCC750. We would also like to mention that our conclusion about the lack of evidence for clonal expansion of fluconazole resistant *C. tropicalis* in Hainan is specific to our sample analyzed here. Indeed, it’s entirely possible that samples from patients and clinics where the use of fluconazole or other triazoles may be prevalent would likely show evidence of such clonal expansion, similar to what has been found in other studies.

In conclusion, the establishment of a MLST database for *C. tropicalis* has facilitated comparisons of strains and populations from different laboratories and different geographic regions around the world. Our analyses identified high and novel genetic diversity of *C. tropicalis* in Hainan samples and revealed no evidence of genetic differentiation among the regional population. Our combined analyses of MLST genotype and fluconazole resistance suggested multiple independent origins of fluconazole resistant and dose-dependent strains in Hainan. Our genotypic comparisons revealed evidence for genotype sharing between strains from Hainan Island and those from other regions including Mainland China, Taiwan Island, the Netherlands, and Brazil. The results and data presented here not only provide an understanding of *C. tropicalis* in tropical Asia but also expand the database for future studies of *C. tropicalis* in other regions. Our study also calls for greater effects in analyzing strains from both clinics and natural environments in the tropics in order to further understand the origins and distributions of fluconazole-resistant genotypes in these regions.

## Materials and Methods

### Isolates

All the samples included in this study were collected from the oral cavities of either healthy people or hospitalized patients (Table 1). All experimental protocols for sampling were approved by Hainan Medical College and informed consent was obtained from all hosts. The sampling procedures were carried out in accordance with relevant guidelines and regulations. The hosts were from seven different cities/municipalities on Hainan Island. The geographical coordinates for the biggest city in each of the seven regions are presented in Table 1. A total of 116 isolates of *C. tropicalis* were obtained in this study, with 23 from health people and 93 from hospitalized patients. However, none of the hosts, including the hospitalized patients, had clinical symptoms of oral thrash at the time of sampling. The procedures for obtaining and identifying the species status of these isolates were described in previous studies^14^,^22^,^42^. The detailed information for each isolate is described in Table 1. The yeast pure cultures were maintained on Sabouraud dextrose broth containing 30% glycerol in −80 °C freezer until use.

### DNA extraction and genotyping

The total genomic DNA of the isolates was extracted using a Yeast DNA miniprep protocol described previously^43^. The DNA concentrations were estimated with a spectrophotometer absorbance at 260 nm and diluted to 10 ng/ml. Each PCR amplification reaction was carried out in a final volume of 50 μl that consisted of 25 μl of 2x Premix Taq (Tiangen), 21 μl of dH_2_O, 2 μl of template DNA, and 2 μl of the forward/reverse primers. The primer sequences and amplification conditions for obtaining sequence information at the six loci (*ICL1, MDR1, SAPT2, SAPT4, XYR1* and *ZWFa1*) followed those described previously^18^. As shown in Supplementary Table 1, these six gene fragments are located on six different chromosomal scaffolds. The amplified fragments were purified using a PCR purification kit (Qiagen) according to the manufacturer’s instructions. Both the forward and reverse strands of the purified DNA fragments were sequenced at the Public Research Laboratory of Hainan Medical College using the same primers as those used in the initial PCR amplification. DNA sequencing was performed with Chromas 2.13 software^44^.

### Sequence type identification at each locus and at the six combined loci

For each locus in each strain, sequence chromatograms from the two directions were aligned using the DNASTAR software (http://www.dnastar.com) to obtain a combined consensus sequence for the locus. Because *C. tropicalis* is a diploid organism, heterozygous nucleotide sites are expected for our isolates. To ensure that all heterozygous sites are accounted for, all sequence chromatograms were manually inspected. These sequences were then compared with the existing sequences at the *C. tropicalis* MLST sequence type database (http://pubmlst.org/ctropicalis/) to obtain a sequence profile for each locus for each strain and a combined diploid sequence type (DST) for each strain based on the sequence profiles at all six gene fragments. The new sequence profiles at both the individual locus and the combined six loci that were absent in the original database were respectively assigned new numbers (Table 1). The sequences for all strains at the six loci have been deposited in the C*. tropicalis* MLST database.

### Relationships among sequence types at each locus and at the combined six loci

To analyze the relationships among our sequences and between ours and those already in the database, we downloaded all the representative sequences for each locus from the *C. tropicalis* MLST database and aligned them together with ours. The sequence relationships at each locus and strain relationships based on sequences at the combined 6 loci were determined through cluster analysis using UPGMA (unweighted pair group method using their arithmetic averages) of the MEGA software^45^. The putative clonal clusters showing the likely ancestor- descendant relationships among the isolates were identified with the eBURST package, v3.0 (http://eburst.mlst.net)^26^.

### Geographic patterns of DNA sequence variation

Since *C. tropicalis* is a diploid yeast and heterozygous nucleotides sites have been frequently found, our analyses of the geographic patterns of DNA sequence variation followed those for diploids. Here, each polymorphic nucleotide site is treated as an informative site and alternative nucleotides at each locus as different alleles. To infer the patterns of genetic variation, the sequences were imported into the computer program GenAlEx 6.5^46^. The population genetic parameters such as the number of polymorphic nucleotide sites within each gene fragment and allelic diversity in each population were estimated. In addition, GenAlEx 6.5 was used to calculate the pairwise population F_ST_ values and determine the potential correlation between genetic and geographical distances (Mantel test). The analysis of molecular variance (AMOVA) was performed to estimate the relative contributions of geographic separation to the overall genetic variation.

### Relationship between fluconazole susceptibility and MLST genotype relatedness

All strains were tested for their susceptibility to fluconazole. The details of antifungal susceptibility testing were described in a previous study^25^. The putative association between genotypes and fluconazole susceptibilities was examined using GenAlEx 6.5. Specifically, we obtained and compared two distance matrices. In one matrix, we obtained the genetic distances between all strain pairs (116 × 115/2 = 6670 pairwise distances) based on the nucleotides at all 79 polymorphic sites. In the second matrix, we obtained the absolute differences in the size (in mm) of the “zone of inhibition” between all strain pairs (also 6670 pairwise distances). A non-parametric Mantel test was used to investigate whether there was a significant correlation between genetic distance and fluconazole susceptibility difference in our sample. In addition, to help visualize the relationships between genetic relationship and fluconazole susceptibility, we also marked S, I, and R respectively beside strains that were susceptible, intermediate, and resistant to fluconazole onto the UPGMA graph representing the genetic relationships among the 116 strains based on sequence information from the six loci.

## Additional Information

**How to cite this article**: Wu, J.-Y. *et al*. Multilocus sequence analyses reveal extensive diversity and multiple origins of fluconazole resistance in *Candida tropicalis* from tropical China. *Sci. Rep.* **7**, 42537; doi: 10.1038/srep42537 (2017).

**Publisher's note:** Springer Nature remains neutral with regard to jurisdictional claims in published maps and institutional affiliations.

## Supplementary Material

Supplementary Information

## Figures and Tables

**Table 1:** Populations of *C. tropicalis* analyzed in this study from Hainan Island of China and their physical geographical information.

Geographic population	Sample size	Latitude	Longitude
Danzhou	20	19.31	109.34
Dongfang	22	19.09	108.64
Haikou	37	19.61	110.72
Wenchang	5	18.14	109.31
Sanya	6	20.02	110.35
Lingshui	21	18.48	110.02
Baoting	4	18.64	109.7
Wuzhishan	1	18.78	109.52

**Table 2:** Information about strains of *C. tropicalis* from Hainan Island.

Strain Name	City/County	Host group	SEX	AGE	*ICL1*	*MDR1*	*SAPT2*	*SAPT4*	*XYR1*	*ZWF1a*	DST	eBURST group
Ct_BT10_hn_Li_m_40_Pt	Baoting	Patient	M	40	1	9	12	14	60	22	430	4
Ct_BT107_hn_F_39_Pt	Baoting	Patient	F	39	9	106	3	23	118	3	498	Singleton
Ct_BT20_hn_m_28_Pt	Baoting	Patient	M	28	1	44	3	7	58	3	149	7
Ct_BT24_hn_Li_F_16_Pt	Baoting	Patient	F	16	3	22	1	10	27	1	487	18
Ct_DZ11_hn_m_2_Pt	Danzhou	Patient	M	2	3	4	1	41	77	4	346	14
Ct_DZ27_hn_m_3_Pt	Danzhou	Patient	M	3	3	22	1	3	9	1	488	18
Ct_DZ29_hn_m_8_Pt	Danzhou	Patient	M	8	1	44	12	7	48	22	330	10
Ct_DZ35_hn_m_28_Pt	Danzhou	Patient	M	28	1	89	1	7	48	9	348	Singleton
Ct_DZ49_hn_m_85_Pt	Danzhou	Patient	M	85	1	7	2	14	100	3	420	11
Ct_DZ50_hn_m_94_Pt	Danzhou	Patient	M	94	3	4	1	41	77	4	346	14
Ct_DZ51_hn_m_92_Pt	Danzhou	Patient	M	92	3	22	1	3	27	1	489	18
Ct_DZ56_hn_m_76_Pt	Danzhou	Patient	M	76	1	9	12	17	85	22	433	4
Ct_DZ65_hn_m_71_Pt	Danzhou	Patient	M	71	1	110	39	33	3	7	466	32
Ct_DZ73_hn_f_29_Pt	Danzhou	Patient	F	29	1	26	3	3	48	3	445	Singleton
Ct_DZ79_hn_f_68_Pt	Danzhou	Patient	F	68	1	90	3	7	9	1	458	51
Ct_DZ85_hn_m_76_Pt	Danzhou	Patient	M	76	1	109	12	17	56	9	465	46
Ct_DZ90_hn_m_90_Pt	Danzhou	Patient	M	90	1	22	3	17	9	22	440	Singleton
Ct_NK136_hn_m_4_Pt	Danzhou	Patient	M	4	1	108	3	3	9	3	463	Singleton
Ct_NK161_hn_f_72_Pt	Danzhou	Patient	F	72	1	53	3	20	23	41	450	Singleton
Ct_NK174_hn_f_54_Pt	Danzhou	Patient	F	54	3	3	3	33	76	4	483	Singleton
Ct_NK191_hn_m_28_Pt	Danzhou	Patient	M	28	1	7	1	7	38	3	419	7
Ct_NK202_hn_f_43_Pt	Danzhou	Patient	F	43	1	111	39	33	3	7	468	32
Ct_NK204_hn_f_90_Pt	Danzhou	Patient	F	90	1	56	1	59	9	3	451	Singleton
Ct_NK211_hn_m_75_Pt	Danzhou	Patient	M	75	1	60	12	7	111	3	454	Singleton
Ct_DFG107_hn_m_55_Pt	Dongfang	Patient	M	55	2	18	5	61	113	5	476	Singleton
Ct_DFG11_sc_m_75_Pt	Dongfang	Patient	M	75	1	9	12	14	60	22	430	4
Ct_DFG118_hn_f_61_Pt	Dongfang	Patient	F	61	1	22	12	17	60	22	331	4
Ct_DFG14_gx_Z_m_58_Pt	Dongfang	Patient	M	58	1	9	12	14	60	22	430	4
Ct_DFG55_sc_m_73_Pt	Dongfang	Patient	M	73	1	9	12	62	60	22	432	4
Ct_DFG56_sc_m_43_Pt	Dongfang	Patient	M	43	1	9	12	62	60	22	432	4
Ct_DFR21_hn_m_54_Pt	Dongfang	Patient	M	54	1	22	1	19	4	7	437	44
Ct_DFR35_hn_m_59_Pt	Dongfang	Patient	M	59	1	9	3	14	60	22	427	4
Ct_DFR36_hn_m_46_Pt	Dongfang	Patient	M	46	1	9	3	14	60	22	427	4
Ct_DFR39_hn_m_8_Pt	Dongfang	Patient	M	8	1	44	12	14	60	22	333	10
Ct_DFR41_hn_Li_m_70_Pt	Dongfang	Patient	M	70	1	4	1	38	72	1	197	2
Ct_DFR46_hn_m_40_Pt	Dongfang	Patient	M	40	2	20	5	61	110	5	479	47
Ct_DFR48_hn_m_59_Pt	Dongfang	Patient	M	59	1	92	1	7	9	1	461	Singleton
Ct_DFR52_hn_f_31_Pt	Dongfang	Patient	F	31	1	22	12	14	60	7	444	4
Ct_DFR77_hn_f_60_Pt	Dongfang	Patient	F	60	1	44	3	7	58	3	149	7
Ct_DFT34_hn_f_52_Pt	Dongfang	Patient	F	52	1	117	3	7	9	32	475	Singleton
Ct_DFT4_hn_m_75_Pt	Dongfang	Patient	M	75	18	115	1	17	9	1	480	Singleton
Ct_DFT6_hn_m_8_Pt	Dongfang	Patient	M	8	1	118	38	14	2	22	464	Singleton
Ct_DFT81_hn_f_1_Pt	Dongfang	Patient	F	1	1	109	12	17	56	9	465	46
Ct_DFT87_hn_f_47_Pt	Dongfang	Patient	F	47	1	22	3	7	72	7	439	23
Ct_DFZ111_hn_f_55_Pt	Dongfang	Patient	F	55	3	114	1	3	27	1	495	18
Ct_DFZ125_hn_f_42_Pt	Dongfang	Patient	F	42	3	117	1	10	1	22	496	Singleton
Ct_1_HNHK_m_91_Pt	Haikou	Patient	M	91	9	90	3	10	117	3	497	Singleton
Ct_11_HNHK_m_63_Pt	Haikou	Patient	M	63	1	9	3	17	85	7	425	Singleton
Ct_12_HNHK_f_25_Pt	Haikou	Patient	F	25	1	113	12	19	116	1	471	Singleton
Ct_13_HNHK_m_43_Pt	Haikou	Patient	M	43	3	61	1	38	77	4	493	Singleton
Ct_14_HNHK_f_32_Pt	Haikou	Patient	F	32	3	22	3	38	9	22	490	Singleton
Ct_15_HNHK_m_76_Pt	Haikou	Patient	M	76	1	22	12	17	60	22	331	4
Ct_16_HNHK_f_25_Pt	Haikou	Patient	F	25	1	112	36	7	68	3	470	48
Ct_2_HNHK_m_79_Pt	Haikou	Patient	M	79	1	9	12	17	60	3	428	4
Ct_26_HNHK_w_79_Pt	Haikou	Patient	F	79	1	9	12	17	2	22	336	4
Ct_27_HNHK_m_52_Pt	Haikou	Patient	M	52	1	3	3	10	3	3	416	Singleton
Ct_29_HNHK_f_76_Pt	Haikou	Patient	F	76	1	17	1	10	9	1	436	Singleton
Ct_3_HNHK_f_90_Pt	Haikou	Patient	F	90	1	9	12	17	60	22	394	4
Ct_30_HNHK_m_38_Pt	Haikou	Patient	M	38	1	44	3	7	58	3	149	7
Ct_31_HNHK_f_76_Pt	Haikou	Patient	F	76	1	9	1	10	54	3	477	Singleton
Ct_32_HNHK_m_62_Pt	Haikou	Patient	M	62	1	22	12	17	60	7	441	4
Ct_33_HNHK_f_79_Pt	Haikou	Patient	F	79	1	22	12	17	60	22	331	4
Ct_4_HNHK_f_65_Pt	Haikou	Patient	F	65	1	9	12	17	60	22	394	4
Ct_5_HNHK_m_69_Pt	Haikou	Patient	M	69	1	22	12	17	53	7	442	4
Ct_6_HNHK_m_66_Pt	Haikou	Patient	M	66	1	9	12	17	26	22	429	4
Ct_7_HNHK_m_60_Pt	Haikou	Patient	M	60	2	20	12	61	60	22	481	Singleton
Ct_8_HNHK_m_59_Pt	Haikou	Patient	M	59	1	7	12	17	24	7	423	Singleton
Ct_9_HNHK_m_57_Pt	Haikou	Patient	M	57	1	9	12	17	60	22	394	4
Ct_HY10_hn_m_53_Pt	Haikou	Patient	M	53	5	61	3	11	68	3	374	53
Ct_HY190_F_75_Pt	Haikou	Patient	F	75	3	51	1	5	77	4	492	Singleton
Ct_HY3__hn_m_67_Pt	Haikou	Patient	M	67	3	22	3	38	9	22	490	Singleton
Ct_HY51_F_63_Pt	Haikou	Patient	F	63	1	9	12	14	60	22	430	4
Ct_HY6_hn_f_56_Pt	Haikou	Patient	F	56	3	3	1	38	76	4	482	Singleton
Ct_HY8_hn_m_75_Pt	Haikou	Patient	M	75	3	7	4	6	52	4	203	1
Ct_SY1_hn_m_46_Pt	Haikou	Patient	M	46	1	58	36	7	68	39	453	Singleton
Ct_SY15_hn_m_52_Pt	Haikou	Patient	M	52	3	107	1	10	115	7	494	Singleton
Ct_SY5_hn_m_89_Pt	Haikou	Patient	M	89	1	89	4	38	94	3	457	Singleton
Ct_SY7_hn_m_74_Pt	Haikou	Patient	M	74	3	42	37	7	114	3	491	Singleton
Ct_SYL_1_4_hn_f_85_Pt	Haikou	Patient	F	85	3	20	5	61	110	5	486	47
Ct_SYL2_1_13_hn_m_72_Pt	Haikou	Patient	M	72	1	61	1	11	4	1	455	34
Ct_SYL3_1_32_hn_m_72_Pt	Haikou	Patient	M	72	1	90	12	7	4	1	460	Singleton
Ct_SYL4_1_39_hn_f_75_Pt	Haikou	Patient	F	75	1	90	3	7	2	1	459	51
Ct_SYL5_2_14_hn_f_65_Pt	Haikou	Patient	F	65	1	44	12	21	94	22	449	10
Ct_C11_HNLS_f_86_HP	Lingshui	Healthy Person (HP)	F	86	1	102	3	11	48	3	462	50
Ct_C13_HNLS_f_80_HP	Lingshui	HP	F	80	1	22	12	17	60	31	443	4
Ct_C14_HNLS_f_13_HP	Lingshui	HP	F	13	38	44	3	7	58	3	501	7
Ct_C16_HNLS_m_43_HP	Lingshui	HP	M	43	1	7	3	7	58	3	421	7
Ct_C17_HNLS_f_60_HP	Lingshui	HP	F	60	1	7	3	7	58	7	422	7
Ct_C18_HNLS_m_26_HP	Lingshui	HP	M	26	1	7	1	7	23	3	418	7
Ct_C2_HNLS_m_10_HP	Lingshui	HP	M	10	1	44	3	7	58	3	149	7
Ct_C20_HNLS_m_18_HP	Lingshui	HP	M	18	38	44	3	7	58	22	502	7
Ct_C21_HNLS_m_18_HP	Lingshui	HP	M	18	1	9	12	17	60	22	394	4
Ct_C22_HNLS_f_20_HP	Lingshui	HP	F	20	1	9	22	17	60	22	434	4
Ct_C23_HNLS_f_86_HP	Lingshui	HP	F	86	1	9	12	17	60	22	394	4
Ct_C25_HNLS_f_41_HP	Lingshui	HP	F	41	1	22	3	7	85	22	438	Singleton
Ct_C28_HNLS_f_57_HP	Lingshui	HP	F	57	1	9	1	3	97	7	351	5
Ct_C29_HNLS_f_60_HP	Lingshui	HP	F	60	1	44	3	7	58	3	149	7
Ct_C3_HNLS_m_19_HP	Lingshui	HP	M	19	1	9	12	17	60	22	394	4
Ct_C30_HNLS_m_10_HP	Lingshui	HP	M	10	3	4	1	41	77	4	346	14
Ct_C33_HNLS_m_83_HP	Lingshui	HP	M	83	1	119	1	17	24	42	478	Singleton
Ct_C34_HNLS_m_39_HP	Lingshui	HP	M	39	1	57	1	23	60	40	452	Singleton
Ct_C36_HNLS_f_56_HP	Lingshui	HP	F	56	1	89	4	60	85	1	456	Singleton
Ct_C38_HNLS_f_66_HP	Lingshui	HP	F	66	1	44	3	7	58	22	446	7
Ct_C4_HNLS_m_11_HP	Lingshui	HP	M	11	1	16	3	7	9	3	23	Singleton
Ct_17_HNSY_m_64_Pt	Sanya	Patient	M	64	5	16	3	7	48	21	343	Singleton
Ct_18_HNSY_f_88_Pt	Sanya	Patient	F	88	1	116	3	38	2	9	473	Singleton
Ct_19_HNSY_m_82_Pt	Sanya	Patient	M	82	1	22	12	17	60	22	331	4
Ct_25_HNSY_f_87_Pt	Sanya	Patient	F	87	1	117	3	4	76	3	474	Singleton
Ct_C26_HNSY_m_17_HP	Sanya	HP	M	17	3	7	1	6	52	3	484	Singleton
Ct_C27_HNSY_m_26_HP	Sanya	HP	M	26	1	22	1	58	52	4	472	Singleton
Ct_20_HNWC_f_30_Pt	Wenchang	Patient	F	30	1	16	1	3	9	1	435	49
Ct_21_HNWC_m_63_Pt	Wenchang	Patient	M	63	1	112	3	7	68	3	469	48
Ct_22_HNWC_m_89_Pt	Wenchang	Patient	M	89	1	9	12	17	60	22	394	4
Ct_23_HNWC_f_30_Pt	Wenchang	Patient	F	30	26	16	1	3	9	1	500	49
Ct_WC91_m_78_Pt	Wenchang	Patient	M	78	1	110	39	33	4	7	467	32
Ct_WZS1_gd_f_42_Pt	Wuzishan	Patient	F	42	1	7	22	10	24	1	424	Singleton

**Table 3:** Comparisons of polymorphisms at the six *C. tropicalis* gene fragments used for MLST among different geographic regions.

Gene loci	US and European countries^17^ (106 isolates) G/P (Ratio)	Brazil^20^ (61 isolates) G/P (Ratio)	Beijing, China^21^ (58 isolates) G/P (Ratio)	Hainan, China (116 isolates) G/P (Ratio)
*ICL1*	15/21 (0.71)	6/20 (0.30)	7/3 (2.33)	8/6 (1.33)
*MDR1*	40/24 (1.67)	17/27 (0.63)	15/6 (2.50)	37/21 (1.76)
*SAPT2*	19/37 (0.51)	8/39 (0.21)	5/2 (2.50)	11/7 (1.57)
*SAPT4*	28/31 (0.90)	8/34 (0.24)	18/13 (1.38)	21/18 (1.17)
*XYR1*	33/11 (3.00)	25/19 (1.32)	15/1 (15.00)	33/16 (2.36)
*ZWFa1*	17/18 (0.94)	8/15 (0.53)	10/3 (3.33)	14/11 (1.27)

(G: Number of genotypes; P: Number of polymorphic nucleotide sites; Ratio: ratio of the number of genotypes over the number of polymorphic nucleotide sites).

**Table 4:** Summary table of AMOVA results.

Source	df	SS	MS	Est. Var.	%	P
Among Regions	2	24.868	12.434	0.000	0%	0.941
Among Populations	4	53.341	13.335	0.196	3%	0.061
Among Individuals	111	973.426	8.770	1.650	23%	0.001
Within Individuals	116	645.500	5.470	5.470	75%	0.001
Total	233	1697.136		7.316	100%	0.001

**Table 5:** Pairwise F_ST_ values between geographic populations of *C. tropicalis* from Hainan.

	Danzhou	Dongfang	Haikou	Wenchang	Sanya
Dongfang	0.033				
Haikou	0.013	0.015			
Wenchang	0.029	0.041	0.028		
Sanya	0.045	0.038	0.023	0.053	
Lingshui	0.022	0.027	0.011	0.042	0.035

Note: None of the pairwise F_ST_ values was statistically significant at P < 0.05.
