# Supplementary material for: Multilocus sequence analyses reveal extensive diversity and multiple origins of fluconazole resistance in Candida tropicalis from tropical China
Source: Sci Rep. 2017 Feb 10;7:42537. doi: 10.1038/srep42537 (PMC5301247; doi:10.1038/srep42537)
Supplement: Supplementary Information [file srep42537-s1.pdf]

Supplementary Information File

Title of Manuscript: Multilocus sequence analyses reveal extensive diversity and multiple origins of fluconazole resistance in *Candida tropicalis* from tropical China

Jin-Yan Wu[1,2], Hong Guo[2], Hua-Min Wang[2], Guo-Hui Yi[2], Li-Min Zhou[2], Xiao-Wen He[2], Ying Zhang[1] and Jianping Xu[1,2,3]*

[1] Laboratory for Conservation and Utilization of Bio-Resources, and Key Laboratory for Microbial Resources of the Ministry of Education, Yunnan University, Kunming, Yunnan, China.

[2] Public Research Laboratory, Hainan Medical University, Haikou, Hainan, China.

[3] Department of Biology, McMaster University, Hamilton, Ontario, L8S 4K1, Canada.

*__Corresponding author:__ jpxu@mcmaster.ca

Short title: *Candida tropicalis* in Hainan, China

Key words: Multilocus sequence typing, novel genotypes, fluconazole resistance, geographic pattern

Supplementary Table S1. Locations of the six analyzed DNA fragments within the assembled genome sequence of *C. tropicalis* strain MYA-3404.

Gene name	Chromosomal scaffold ID	Scaffold length (bp)	Gene location within scaffold (bp)
ICL1	NW_003020058.1	1,255,791	959,343-959789
MDR1	NW_003020055.1	2,216,334	754,510-754086
SAPT2	NW_003020038.1	2,474,448	1,573,960-1,574,484
SAPT4	NW_003020049.1	2,308,670	2,274,975-2,274,586
XYR	NW_003020040.1	419,327	35,519-35,150
ZWF	NW_003020056.1	1,654,078	515,841-516,360

The GenBank accession number for this genome sequence is AAFN01000000. The relevant information about this genome can be accessed at: www.ncbi.nlm.nih.gov/bioproject/13675. The above six scaffolds are among 23 chromosomal scaffolds assembled for this *C. tropicalis* genome.

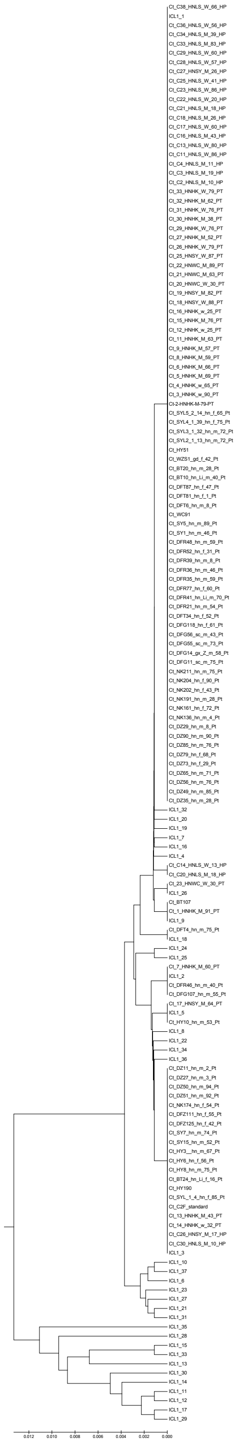

Supplementary Figure S1. Relationships among the unique genotypes at the *ICL1* locus in the *Candida tropicalis* MLST database and those of our 116 strains from Hainan Island.

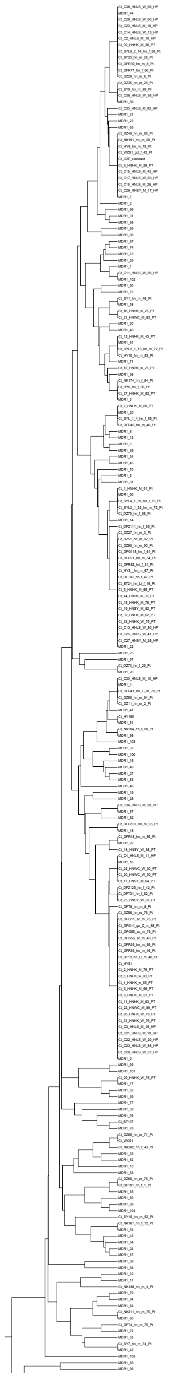

Supplementary Figure S2. Relationships among the unique genotypes at the *MDR1* locus in the *Candida tropicalis* MLST database and those of our 116 strains from Hainan Island.

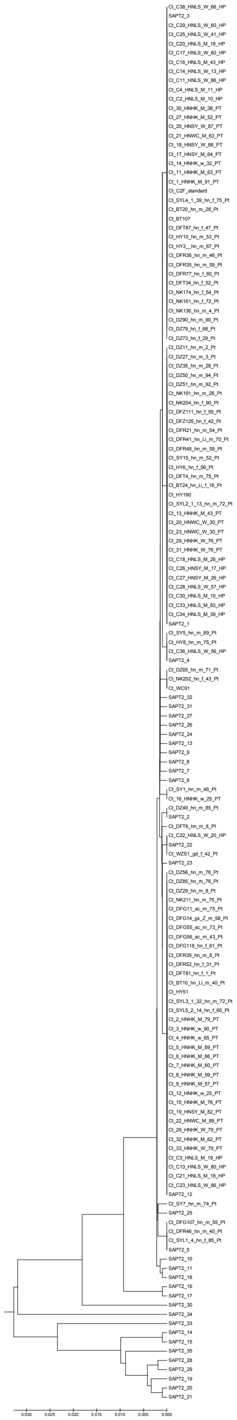

Supplementary Figure S3. Relationships among the unique genotypes at the *SAPT2* locus in the *Candida tropicalis* MLST database and those of our 116 strains from Hainan Island.

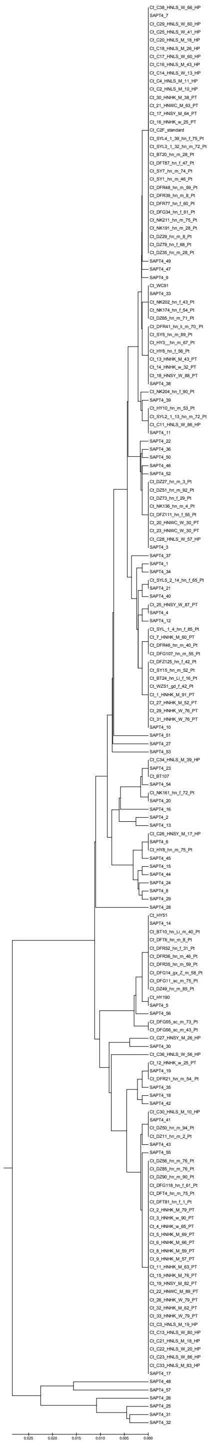

Supplementary Figure S4. Relationships among the unique genotypes at the *SATP4* locus in the *Candida tropicalis* MLST database and those of our 116 strains from Hainan Island.

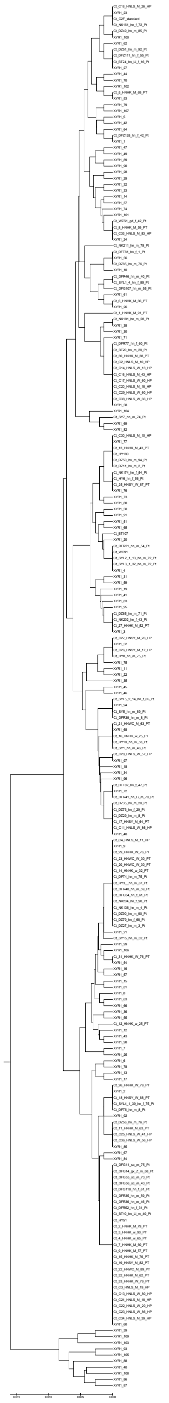

Supplementary Figure S5. Relationships among the unique genotypes at the *XYR1* locus in the *Candida tropicalis* MLST database and those of our 116 strains from Hainan Island.

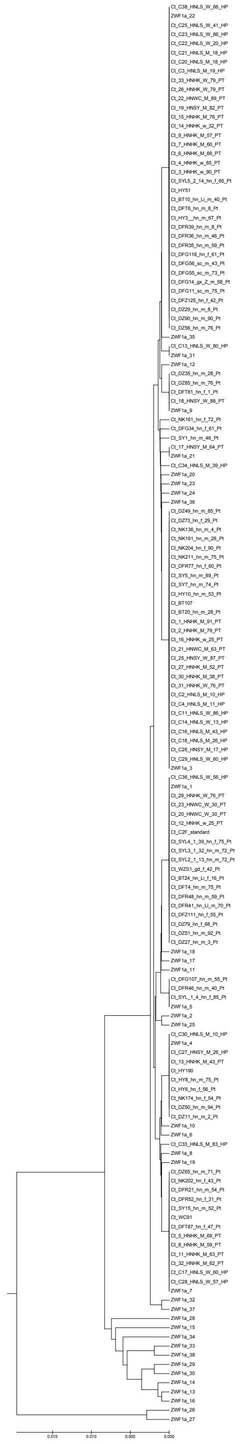

Supplementary Figure S6. Relationships among the unique genotypes at the *ZWF1a* locus in the *Candida tropicalis* MLST database and those of our 116 strains from Hainan Island.
